# Complicated pyelonephritis associated with chronic renal stone disease

**DOI:** 10.1007/s11547-020-01315-7

**Published:** 2020-11-27

**Authors:** Federica Ciccarese, Nicolò Brandi, Beniamino Corcioni, Rita Golfieri, Caterina Gaudiano

**Affiliations:** Department of Radiology, IRCCS Azienda Ospedaliero-Universitaria di Bologna, Via Albertoni 15, Bologna, Italy

**Keywords:** Xanthogranulomatous pyelonephritis, Emphysematous pyelonephritis, Complicated pyelonephritis, Renal stone disease, Nephrolithiasis

## Abstract

**Purpose:**

This article reviews imaging manifestations of complicated pyelonephritis associated with chronic renal stones disease, in particular xanthogranulomatous pyelonephritis (XGP) and emphysematous pyelonephritis (EPN), as potential mimics of other renal diseases and malignances and provides helpful tips and differentiating features that may alert the radiologist to suspect a diagnosis of infection.

**Materials and methods:**

A retrospective review of the records from 6 adult patients (5 females and 1 male, mean age 72,3 years) with diagnosis of XGP associated with chronic nephrolithiasis and 7 adult patients (6 females and 1 male, mean age 59,3 years) with diagnosis of EPN associated with chronic nephrolithiasis from January 2010 to January 2020 was carried out. Computed tomography urography (CTU) with at least an unenhanced scan, and the parenchymal and excretory phases after contrast medium administration performed at our Teaching Hospital were included. When available images related to conventional radiography, ultrasound (US) and magnetic resonance imaging of the same patients, the comparison with CTU images was carried out.

**Conclusion:**

A possible diagnosis of XGP or EPN must always be taken into account when a pyelonephritis is associated with untreated kidney stones, especially whenever clinical presentation is atypical, current therapy is not effective and imaging shows features of dubious interpretation. Due to their rarity and atypical presentation, a multidisciplinary approach is required and an expert radiologist represents a key figure in the multidisciplinary team as he can help to differentiate between benign and malignant lesions and thus avoid unnecessary radical surgical procedures.

## Introduction

Renal stone disease is very common, affecting 5–20% of worldwide population, and its prevalence is increasing in industrialized countries, due to dietary factors and even global warming [1]. Nephrolithiasis is now recognized as both chronic and systemic conditions [2], further underscoring the great impact of the disease and the consequent economic burden on the healthcare system [3]. In fact, several studies have proved that stone formers’ morbidity and mortality rates are higher compared to control subjects, independently from comorbidities associated such as diabetes, heart failure and hypertension.

Chronic renal stone disease is a major risk factor for urinary tract infection and, when the latter overlaps, pyelonephritis may occur, leading to a progressive renal function deterioration and, occasionally, end-stage kidney [4]. In some cases, if left untreated, pyelonephritis can develop complications and evolve into the rare xanthogranulomatous pyelonephritis (XGP) or the life-threatening emphysematous pyelonephritis (EPN) [5]. On top of that, local inflammatory and irritative changes, induced by the persistence of kidney stones, can contribute to epithelium dedifferentiation and dysplasia and thus favor cancer development, rising its chance of occurrence more than twice [6].

Generally, the diagnosis of pyelonephritis is simple, based on clinical and laboratory characteristics. However, given the wide spectrum of complications that can arise from nephrolithiasis, discriminating between benign conditions and malignancies in cases of complicated pyelonephritis may be difficult, unless nephrectomy is performed. For this reason, XGP and EPN still constitute a challenging diagnosis for clinicians, because they are either rare and poorly known, or difficult to identify and discriminate from other chronic kidney diseases [7]. Imaging, even if not routinely required, can play a fundamental role in their diagnosis, especially when the clinical presentation is atypical, in patients who do not respond to therapy or in life-threatening cases, to confirm the diagnosis and define disease’s localization and extension [8]. Moreover, given that complicated pyelonephritis, especially XGP in its focal form, can mimic or even be associate with renal cancer [9], the combined analysis of ultrasound (US), computed tomography (CT) and magnetic resonance imaging (MRI) features plays a useful role in achieving a correct preoperative diagnosis and setting up proper management. Furthermore, since urine cytology is often nonspecific and kidney biopsy is invasive and also accompanied by the risk of spreading the tumor or the infection through the needle tract, radiologists can suggest a diagnosis of complicated pyelonephritis with a potentially benign course, avoiding unnecessary radical therapy [8].

Here, we present a case series of 13 patients with complicated pyelonephritis, in particular XGP and EPN, associated with chronic renal stones disease as potential mimics of other renal diseases and malignancies, along with a review of the literature; moreover, this article provides helpful tips and differentiating features that may alert the radiologist to suspect a diagnosis of infection.

## Materials and methods

A retrospective review of the records from 6 adult patients (5 females and 1 male, mean age 72,3 years) with diagnosis of XGP associated with chronic nephrolithiasis and 7 adult patients (6 females and 1 male, mean age 59,3 years) with diagnosis of EPN associated with chronic nephrolithiasis from January 2010 to January 2020 was carried out.

All six patients with suspected CT images for XGP underwent nephrectomy with subsequent pathological sampling confirming the diagnosis; however, two of them were affected also by a concomitant renal cell carcinoma, which had not been suspected during the CT examination. Only one of the seven patients with CT suspected for ENP underwent biopsy, due to the severity of his clinical–radiological picture, which, however, excluded the presumptively advanced diagnostic hypothesis of pyelonephritis and instead confirmed the diagnosis of massive renal infarction. Two of the patients with a presumptive diagnosis of ENP had previously undergone stent procedures, which, in hindsight, proved to be the cause of the detected gas collections. In this analysis, only CT urography (CTU) with at least an unenhanced scan, and the parenchymal and excretory phases after contrast medium administration performed at our Teaching Hospital were included. On an independent workstation, the CTU images were carefully reviewed in order to identify all signs of XGP and ENP. Moreover, reformatted images (MPR and MIP) were generated searching for the typical changes in the urinary tract related to chronic pyelonephritis. Display windows for the lungs were also used to allow a detailed analysis of the gas collections in the urinary tract in suspected ENP cases. When available images related to conventional radiography, US and MRI of the same patients, the comparison with CTU images was carried out. The pathological findings were then correlated with the radiological findings and with literature data.

The study is an observational, retrospective, single-center study and was approved by our local institutional review board (IRB). All patients provided informed written consent for the procedure and for the processing of personal data.

## Xanthogranulomatous pyelonephritis (XGP)

XGP is a particularly rare form of chronic granulomatous pyelonephritis, mainly affecting females with a 3:1 ratio [10]. Even if it typically affects adults, there are several reports of XGP occurring in children, where must always be differentiated from the more common Wilms’ tumor [11].

The disease has a subtle, often asymptomatic, course and can be accompanied only by discomfort, weight loss and mild fever, with nonspecific laboratory changes such as anemia and leukocytosis; however, it remains a serious condition that, if not treated, can progressively lead to kidney failure over time [12].

XGP pathogenesis is still not very clear, although it can probably be the result of chronic obstruction and infection. The most frequently involved microorganisms are *Proteus mirabilis* and, secondly, *Escherichia coli*, with positive urine cultures in over 60% of cases [13]. However, since obstruction and infection are common urological problems and XPG occurs so infrequently, other factors must be involved in the development of this disease. In particular, once the pathological process has been established, parenchymal destruction occurs with the replacement of the normal renal parenchyma by foamy macrophages called xanthocytes, characterized by a high content of lipids. For this reason, an abnormal lipid metabolism as well as an incomplete immune reaction secondary to a subacute infectious process has been considered as possible etiologic factors in XPG, but there is no experimental evidence to support this theory [14].

XGP is generally unilateral and its diffuse form is reported in most of the cases (90%), with possible extension to peri- and para-renal tissues and to the retroperitoneum; its less common localized form (10%) presents itself as circumscribed swelling and, thus, can mimic renal cancer [15].

**Fig. 1:**
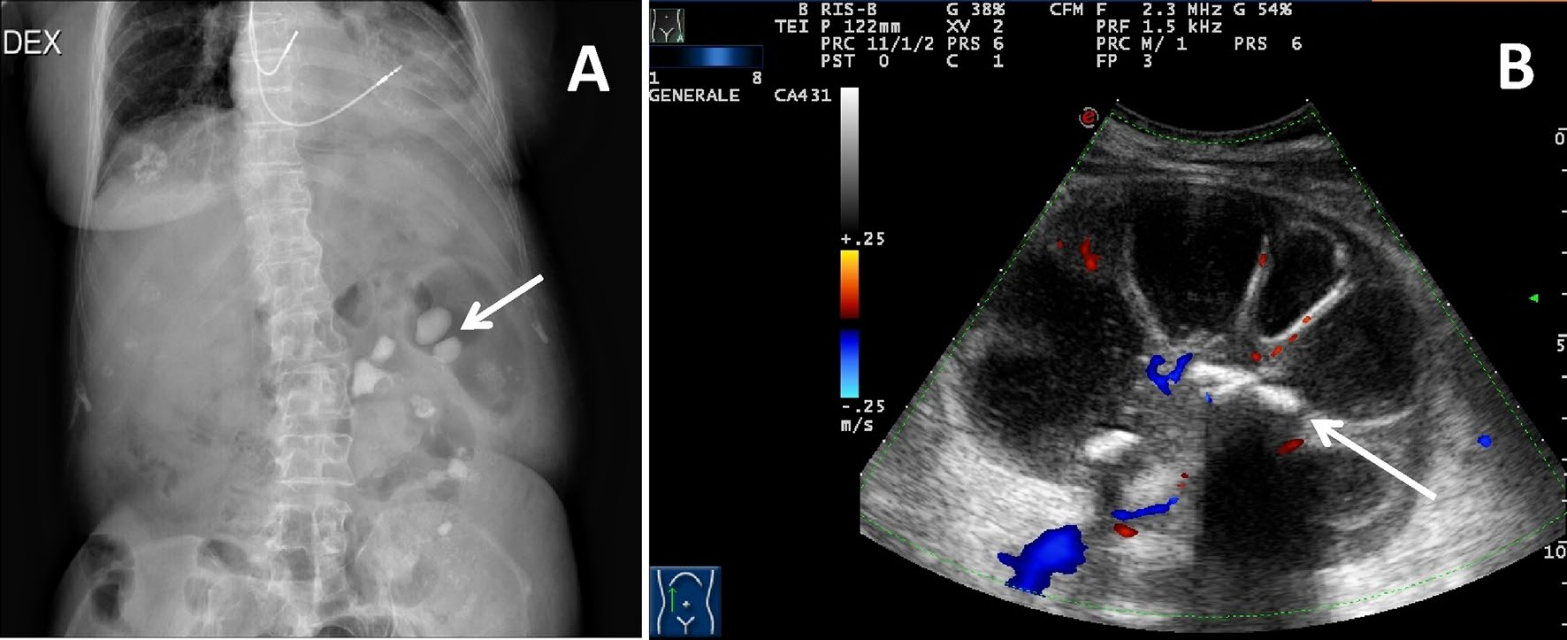
Radiographic and ultrasonographic (US) appearance of xanthogranulomatous Pyelonephritis. A 69-year-old female patient presenting with right flank pain. Conventional radiography (**a**) shows the presence of several big radiopaque staghorn calculus in the left kidney (arrow) and an enlargement of the renal outline. US (**b**) shows an enlarged left kidney with a loss of the normal renal architecture due to the cortical thinning and the presence of multiple hypoechoic areas in the parenchyma, corresponding to dilated calyxes; the renal pelvis is occupied by multiple amorphous echogenicities with acoustic shadowing corresponding to a staghorn calculus (arrow); Doppler US examination demonstrates no particular alterations in the vascularization of the organ

Conventional radiological examination does not provide much information for diagnosis, although it may show the presence of some big radiopaque staghorn calculus in most, but not all, cases; an additional radiographic finding is represented by the enlargement of the renal outline (Fig. 1a) [16].

US is also quite nonspecific, showing an increase in the kidney size, the presence of a single or multiple echogenicities corresponding to renal pelvis staghorn calculus and possible hydronephrosis; however, it is possible to observe a more or less diffuse parenchymal inhomogeneity, with multiple hypoechoic areas (corresponding to the dilated calyxes) due to the loss of the normal renal architecture. Contrast-enhanced US (CEUS) shows no particular alterations in the vascularization of the organ. It is important to remember that the localized form can present itself as a hypoechoic solid mass with an echogenic cystic echoes, similarly to some renal tumors (Fig. 1b) [17].

**Fig. 2:**
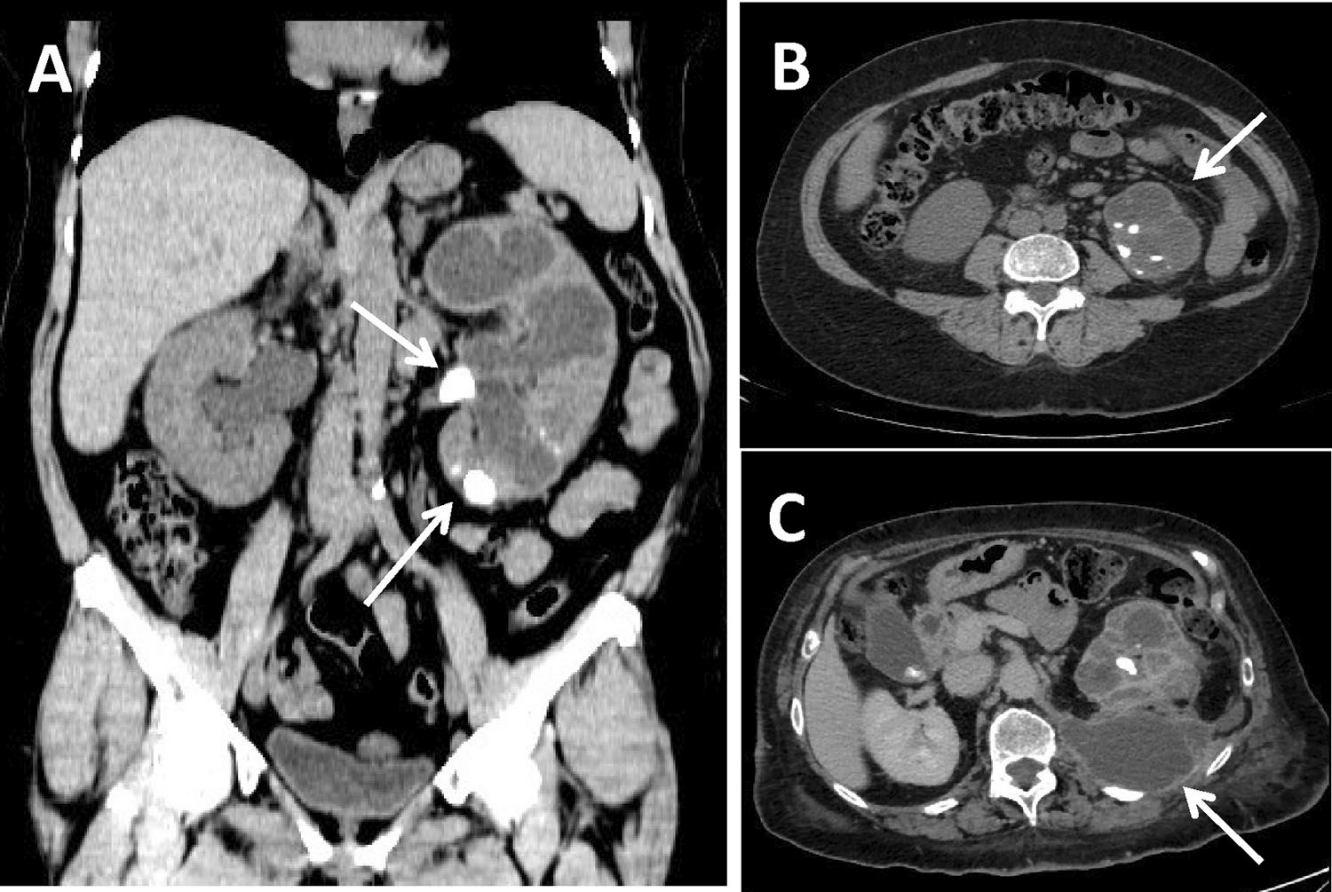
CT staging of xanthogranulomatous pyelonephritis. **a** A 76-year-old female patient presenting with mild fever and stage I XGP with multiple voluminous high-density staghorn calculi in the contracted renal pelvis and inferior calyxes (arrows), concomitant calyx dilatation and cortical thinning. **b** A 68-year-old male patient presenting with weight loss and mild fever and stage II XGP where, besides the presence of multiple hypodense areas of xanthogranulomatous tissue inside an inhomogeneous parenchyma, is evident a thickening of the Gerota fascia with streaks of perirenal fat (arrow) and enlarged lymph nodes at the renal hilum and in the para-aortic region. **c** A 82-year-old female patient presenting with pain, fever and hematuria and severe stage III XGP with a voluminous fluid collection bounded by vascularized tissue in the retroperitoneal space involving the psoas muscle (arrow)

CTU is still the gold standard for the diagnosis of XPG, being able to identify, in the context of an inhomogeneous parenchyma, the presence of multiple hypodense areas of xanthogranulomatous tissue, whose density is variable from − 15 to + 30 HU, depending on the proportion of lipids. Xanthogranulomatous areas appear surrounded by an enhancement rim and, sometimes, may contain calcifications, especially in advanced cases. As mentioned before, XGP is often associated with kidney stone disease so the presence of a single or multiple staghorn calculus filling the calyces is quite common [15]. Recently, the association of calyx dilation, pelvic contraction and cortical reduction has been described as the bear’s paw sign, which represents a strong index of suspicion for the diagnosis of XGP. CT also allows to evaluate the possible presence of hydro-pyonephrosis, the thickening of the Gerota fascia (associated or not with streaks of perirenal fat), retroperitoneal and psoas involvement [18]. Malek and Elder [19] classified this disease into three stages according to the extent of tissue involvement (Table 1): stage I, confined to renal parenchyma only (Fig. 2a), stage II, the most common, involving perinephric fat along with renal parenchyma (Fig. 2b), and stage III, extending into retroperitoneum with involvement of adjacent structure such as psoas muscle (Fig. 2c).

**Table 1:** CT staging of xanthogranulomatous pyelonephritis

Classification	Cass
Malek and Elder (19)	I. Inflammation confined to renal parenchyma only (nephric XGP)
	II. Inflammation involves both the renal parenchyma and the perirenal fat (perinephric XGP)
	III. Inflammation involves the renal parenchyma, the perirenal fat and the retroperitoneum (paranephric XGP)

**Fig. 3:**
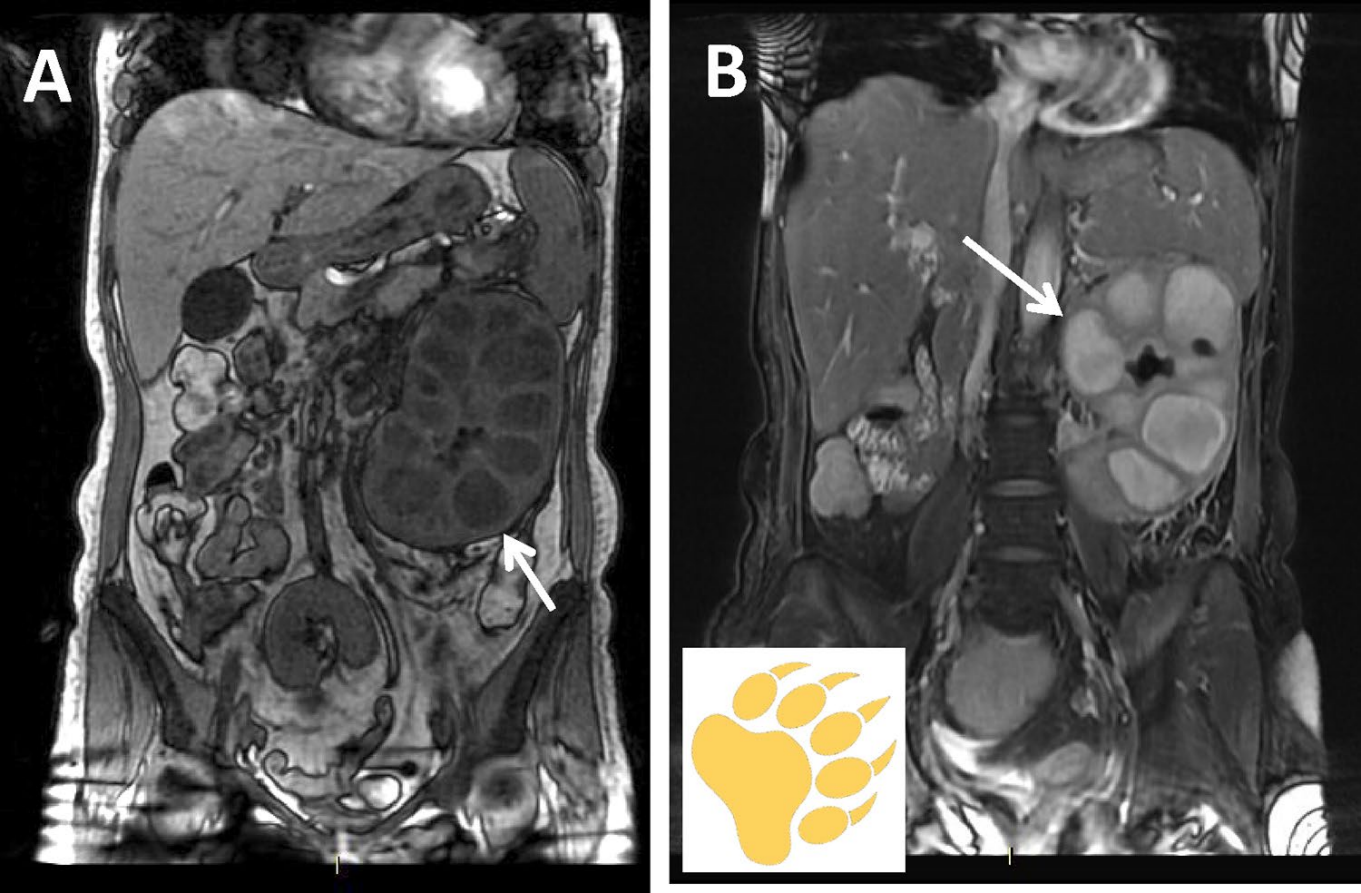
MRI appearance of xanthogranulomatous pyelonephritis. A 75-year-old female patient presenting with flank pain and fever. **a** Coronal T1-weighted images shows hypointense parenchymal cavities (arrow). **b** Coronal T2-weighted sequence shows hyperintense cavities (arrow) surrounded by a solid isointense rim and containing fluid levels, with upper more hyperintense component and lower more hypointense component, due to inflammatory debris and necrotic-purulent material; the combination of contracted renal pelvis, calyx dilatation and cortical thinning together constitutes the bear’s sign

MRI, thanks to its multiparametric evaluation and its excellent characterization of the tissue lipid component, is especially useful in the differential diagnosis between focal form and renal carcinoma and in the evaluation of the extension of the disease to adjacent tissues [20, 21]. The T1-weighted sequences show hypointense fluid cavities surrounded by an iso-hyperintense solid rim, variable based on the percentage of xanthogranulomatous cells, comparable to subcutaneous fat. On the contrary, at T2-weighted sequences, the cavities had a slightly low signal intensity, suggesting a very high protein content, and are surrounded by a solid isointense rim, comparable to the healthy renal parenchyma; fluid levels can be distinguished within the cavities, with upper more hyperintense component and lower more hypointense component (for the heterogeneity of their content, consisting of fluid, debris and pus) (Fig. 3a, b). Moreover, the presence of foamy lipid macrophages can result in signal abatement in fat-suppressed sequences or out-of-phase FSPGR T1-weighted images [22]. After the administration of contrast medium, an enhancement of the rim is appreciated, due to the contextual inflammatory hypervascularization. The infiltration of perirenal tissues appears hypointense in both T1 and T2 sequences, probably due to its partially fibrotic content [23]. Recent studies showed that DWI could represent an adjunctive tool for investigating suspected XGP, since it documented low apparent diffusion coefficient (ADC) values at the central portions of the xanthogranulomatous cavities, attributable to their purulent content [21].

Since MRI has a low sensitivity in detecting kidney stones, it is a prerogative to integrate this investigation with a CT scan, which instead represents the gold standard for their detection, being also able to demonstrate absent opacification of the urinary tract of the affected kidney during the excretory phase.

This disease has been called the “great imitator” because the clinical and radiological findings closely resemble other pathological entities such as renal cell carcinoma, especially focal XGP, and its preoperative diagnosis is often underestimated or incorrect [13, 16, 19]. Moreover, XGP can invade adjacent structures, which makes it even more difficult to distinguish from an aggressive malignancy [24]. However, efforts should be made to establish a correct preoperative diagnosis of XGP in order to save an unnecessary radical surgical intervention, especially in those patients with a partially functioning kidney.

### Differential diagnosis with renal carcinoma

Differential diagnosis with clear cell renal carcinoma, the most common histological variant (75% of cases), can be easily achieved with CTU, since it is usually characterized by an intense contrast uptake in the arterial phase (120–140 HU) and a typical washout in the nephrographic phase (90–100 HU); at MRI, clear cell carcinoma presents a signal intensity similar to the one of the renal cortex at T1-weighted images, while in T2 sequences it is generally hyperintense [25]. However, when a clear cell carcinoma is suspected, a diagnosis of XGP is generally suggested because of the more triangular-shaped aspect of the parenchymal defect on CT and because of the low-signal intensity on fast T2-weighted sequences, differing from the hyperintensity of the clear cell carcinoma [26, 27].

The distinction between XGP and the hypovascular subtypes of renal carcinoma is, however, more complex, especially because both papillary and chromophobe carcinomas tend to appear more homogeneous at CTU and MRI compared to the adjacent renal parenchyma and clear cell carcinoma [28]. In particular, in the arterial phase at CT, these tumors present only a moderate contrast uptake, respectively, of 50–60 HU for papillary carcinoma and 80–100 HU for the chromophobe variant, with a progressive uptake in the nephrographic phase, respectively, of 65–75 HU and 120–130 HU. At MRI, papillary carcinoma frequently appears markedly hypointense on T2-weighted images, probably due to its hemosiderin content and sometimes can contain internal foci of fat that, even in out-of-phase sequences, makes it difficult to distinguish from XGP [29]. Instead, chromophobe carcinoma tends to present a slight hypo-isointensity at T2 sequences, occasionally with a central scar [30]. However, fortunately, the comparative analysis of the contrast uptake by tumors at multiphase MRI examinations (cortico-medullary, nephrographic and excretory phases) represents an effective tool for renal carcinoma’s characterization, showing significantly higher values in clear cell subtype (230%, 250% and 227%, respectively) than in papillary (49%, 92% and 88%, respectively) or in chromophobe variants (98%, 183% and 159%, respectively) [31]. These features can be very useful in differentiate XGP from hypovascular malignancies since the long-standing hypointensity of the mass compared to enhancing renal cortex during the whole course of the dynamic contrast-enhanced MRI study can rule out renal carcinoma, which on the contrary shows an increased signal [32]. DWI can be a useful tool in differentiating between XGP and renal cell carcinoma, demonstrating a more marked restricted diffusion in the central portion of the xanthogranulomatous cavities, compatible with their inflammatory content, compared to the moderate restriction of tumor’s solid regions or the free diffusion of its cystic portions; moreover, ADC values of inflammatory lesions were significantly lower than those of renal cell carcinoma [33]. The CT and MRI features of focal XGP and the most common subtypes of renal cell carcinoma are summarized in Table 2.

**Table 2:** CT and MRI features of focal XGP and the most common subtypes of renal cell carcinoma

	CT characteristics	MRI characteristics
Focal XGP	Hypodense mass (density variable from − 15 to + 30 HU) Enhancement rim Staghorn calculus Bear’s paw sign Possible extension into retroperitoneum (with psoas involvement)	Hypointense mass with iso-hyperintense rim in T1-weighted images Slightly hyperintense mass with isointense rim in T2-weighted images Signal abatement in fat-suppressed sequences Marked restricted diffusion in the central portion and significantly low ADC values in DWI sequences
Clear cell renal carcinoma	Heterogeneously isodense mass Intense contrast enhancement in the arterial phase (120–140 HU) with a progressive washout in the nephrographic phase (90–100 HU)	Isointense mass in T1-weighted images Hyperintense mass in T2-weighted images Intense contrast enhancement (230% in cortico-medullary phase, 250% in nephrographic phase and 227% in excretory phase) Restricted diffusion and moderately low ADC values in DWI sequences; free diffusion in correspondence of cystic areas
Papillary renal carcinoma	Heterogeneously isodense mass Moderate contrast enhancement in the arterial phase (50–60 HU) with a progressive uptake in the nephrographic phase (65–75 HU)	Isointense mass in T1-weighted images Marked hypointense mass in T2-weighted images Occasionally can contain internal foci of fat characterized by signal abatement in fat-suppressed sequences Moderate contrast enhancement (49% in cortico-medullary phase, 92% in nephrographic phase and 88% in excretory phase) Restricted diffusion and moderately low ADC values in DWI sequences
Chromophobe renal carcinoma	Heterogeneously isodense mass Moderate contrast enhancement in the arterial phase (80–100 HU) with a progressive uptake in the nephrographic phase (120–130 HU)	Isointense mass in T1-weighted images Slightly hypointense mass in T2-weighted images Occasionally with a central scar Moderate contrast enhancement (98% in cortico-medullary phase, 183% in nephrographic phase and 159% in excretory phase) Restricted diffusion and moderately low ADC values in DWI sequences

FDG-PET/CT generally cannot distinguish renal inflammation diseases from various types of tumors [34], and the role of fine-needle aspiration cytology (FNAC) is limited due to the great resemblance between the cells of XGP and those of renal carcinoma; thus, definitive diagnosis is usually made following renal biopsy or nephrectomy [35].

**Fig. 4:**
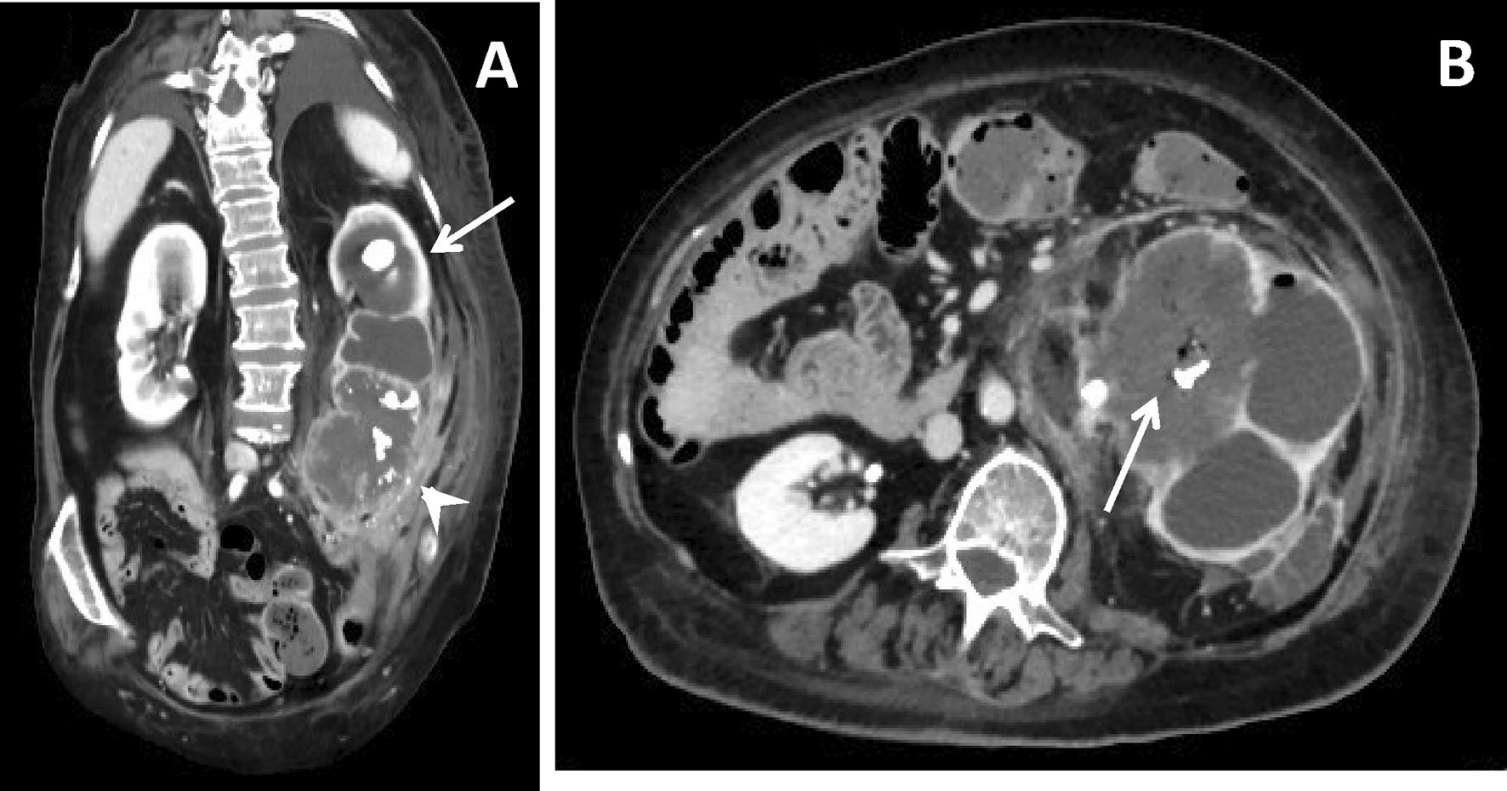
CT of the xanthogranulomatous pyelonephritis associated with renal tumor. A 64-year-old female patient presenting with abdominal pain, fever and hematuria. Coronal oblique MPR reconstruction (**a**) and axial scan (**b**) of the parenchymal phase of an unusual and rare case of XGP associated with high-grade squamous cell carcinoma (G3, pT4), histopathologically confirmed. The upper portion of the kidney is characterized by multiple hypodense areas of xanthogranulomatous tissue and voluminous high-density staghorn calculi (arrow in A); the lower pole shows a complete and severe distortion of the parenchyma, with several calcifications (arrowhead in A and arrow in B), that turned out to be with a carcinomatous degeneration. Several adjacent structures are invaded by the pathological process

It is important not to forget that there are numerous reports in the literature describing simultaneously coexistence of XGP with renal carcinoma, since both these conditions are associated with long-standing, severe, chronic stones and infections (Fig. 4a, b). In fact, it is postulated that chronic irritation of the renal pelvis results in squamous metaplasia, which later increases the risk of developing into cancer [36]. Since metaplasia is known to occur in association with calculi, its presence along with XGP is not surprising, even though only isolated incidence of such lesion is described in the literature, where moreover the metaplasia had already developed into squamous cell carcinoma [35]. Anyway, whether the occurrence of simultaneous metaplasia and XGP is due to the presence of the same long-standing calculus that favors both conditions at the same time or is rather due to a renal carcinoma protruding into the renal pelvis and obstructing the urinary tract, leading to a subsequent XGP, is not clear yet [37].

### Differential diagnosis with renal tuberculosis

Other inflammatory conditions can mimic XPG, and in particular, renal tuberculosis can easily be mistaken for it, due to the very similar thickening of the perirenal fasciae and the common spreading of inflammation into the adjacent organs [38]; US, CTU and MRI findings can help in these cases, showing papillary necrosis, calyceal ulceration, infundibular stenosis and/or dystrophic parenchymal calcifications typical of tuberculosis, as well as other typical lesions in other organs besides the kidney [39].

### Differential diagnosis with lymphoma and leukemia

In most cases, renal lymphoma represents an extranodal spread of a non-Hodgkin’s lymphoma, while primary form, with no other systemic manifestation, is rare. Generally, renal lymphoma appears as multiple renal masses (60% of cases) and less frequently as solitary lesion or as diffuse parenchymal infiltration. In diffuse form, nephromegaly could be the only CT finding, with preservation of cortical profile and deformity of the calyces and pelvis; after contrast administration, infiltrative lymphomatous involvement appears hypodense compared with the normal parenchyma, with evidence of loss of corticomedullary differentiation [18].

Renal involvement from leukemia closely mimics radiological findings of renal lymphoma, appearing likewise as multiple hypodense lesions in an enlarged kidney [40].

### Differential diagnosis with other renal diseases

Renal angiomyolipoma is a benign neoplasm composed of a variable mixture of adipose tissue, blood vessels and muscles that can mimic focal XGP, especially in case of low lipid content; moreover, these tumors can show variable degrees of enhancement depending on the amount of their vascularized tissue components [41].

In children, the differential diagnosis of XGP includes intrarenal Wilms’ tumor and extrarenal neuroblastoma. On CT, these pediatric malignancies appear heterogeneous and, after contrast administration, generally enhance less than the adjacent renal parenchyma; encasement of vessels, a paravertebral location and spinal canal invasion are highly suggestive of neuroblastoma [42]. On MRI, Wilms’ tumor demonstrates a heterogeneous hypointense signal on T1 sequences and appears hyperintense on T2 sequences [43].

Sarcoidosis is a systemic disorder of unknown origin that can affect the kidney as well as the lung, skin and other parenchymal organs; thus, it must be differentiated from XGP. At contrast-CT, renal involvement may manifest as interstitial nephritis, producing a suggestive striated nephrogram; moreover, multiple isodense masses with poor enhancement can be observed, often associated with similar nodules in other parenchymal organs and the lungs [44]. At MRI, sarcoidosis tends to remain isointense compared to surrounding renal parenchyma both in T1 and in T2 [45].

Malakoplakia is a rare granulomatous inflammatory disease secondary to an inadequate intracellular killing of bacteria by histiocytes, presenting with typical intracellular inclusions called Michaelis–Gutmann bodies [46]. Even if the classic appearance is an enlarged kidney with multiple hypovascular nodules, characterized by a poor enhancement at contrast-CT, sometimes malakoplakia may manifest as a bulky mass distorting the renal parenchyma, mimicking focal XGP; at MRI, the latter appears as multifocal hypointense masses at both T1 and T2 sequences [47].

## Emphysematous pyelonephritis (EPN)

EPN is an uncommon form of acute necrotizing pyelonephritis, mainly affecting adult females. The disease has a potentially fatal course, with mortality of 40–90%, and symptoms such as dysuria, fever, nausea, vomiting, pain, and even loss of consciousness.

EPN is typically associated with diabetes mellitus (almost 95% of cases) [47], but chronic urinary stasis and nephrolithiasis are also frequently described in these patients.

The disease is characterized by accumulation of gas within the renal parenchyma, the collecting system as well as the perinephric tissue. However, its pathogenesis remains still obscure, even if several reports claim that it could probably involve four principal factors, namely gas-forming microorganisms, high tissue glucose levels, impaired tissue perfusion and reduced host immunity, all factors that feature prominently in patients with diabetes mellitus [48]. In particular, it has been postulated that the high tissue glucose levels can provide a substrate for microorganisms able to produce CO_2_ and H_2_ by fermentation, both through mixed acid fermentation, such as *Escherichia coli*, *Klebsiella pneumoniae* and *Proteus mirabilis*, and through butyric fermentation of glucose, such as *Clostridium septicum*. The trace amounts of NH_3_ and methane reported in the literature could arise from the degradation of necrotic tissue and the fermentation of amino acids [49].

Conventional radiological examination can show an abnormal gas shadow in the renal bed in about 30% of cases, raising suspicion of EPN. However, US and especially CTU are certainly more valuable since they can easily confirm the presence of intrarenal gas [50]. In particular, US usually demonstrates an enlarged kidney with hyperechoic gas collections inside the parenchyma and/or the collector system [48]. The hyperechogenic foci appear different than those seen in typical renal stone disease, with the distal shadowing having “ring-down” artifacts from reverberation [51] and low-level echoes that are known as “dirty shadowing” [47].

The CTU is the imaging procedure of choice because it can identify, in the context of an enlarged kidney, the presence of multiple collections of gas dissecting the interstitial space, determining infection extension. Moreover, CT scan represents the gold standard for calculus detection, often associated with EPN, and can help to detect eventual urothelial obstruction.

In EPN, renal parenchyma appears inhomogeneous, studded with multiple hypodense fluid-gaseous abscesses (ranging from 0 to 30 UH, depending on the necrotic content) characterized by a rim of enhancement and filled with gas bubbles [48].

In 1996, Wan et al. [52] categorized EPN patients into two groups based on CT findings, with class I referring to those with parenchymal destruction with either the absence of fluid collection or presence of streaky or mottled gas and class II referring to those with either renal or perinephric collection with bubbly or located gas or gas in the collecting system; class 1 is associated with a worse prognosis and a mortality of 70% (versus mortality of 20% in class 2).

**Fig. 5:**
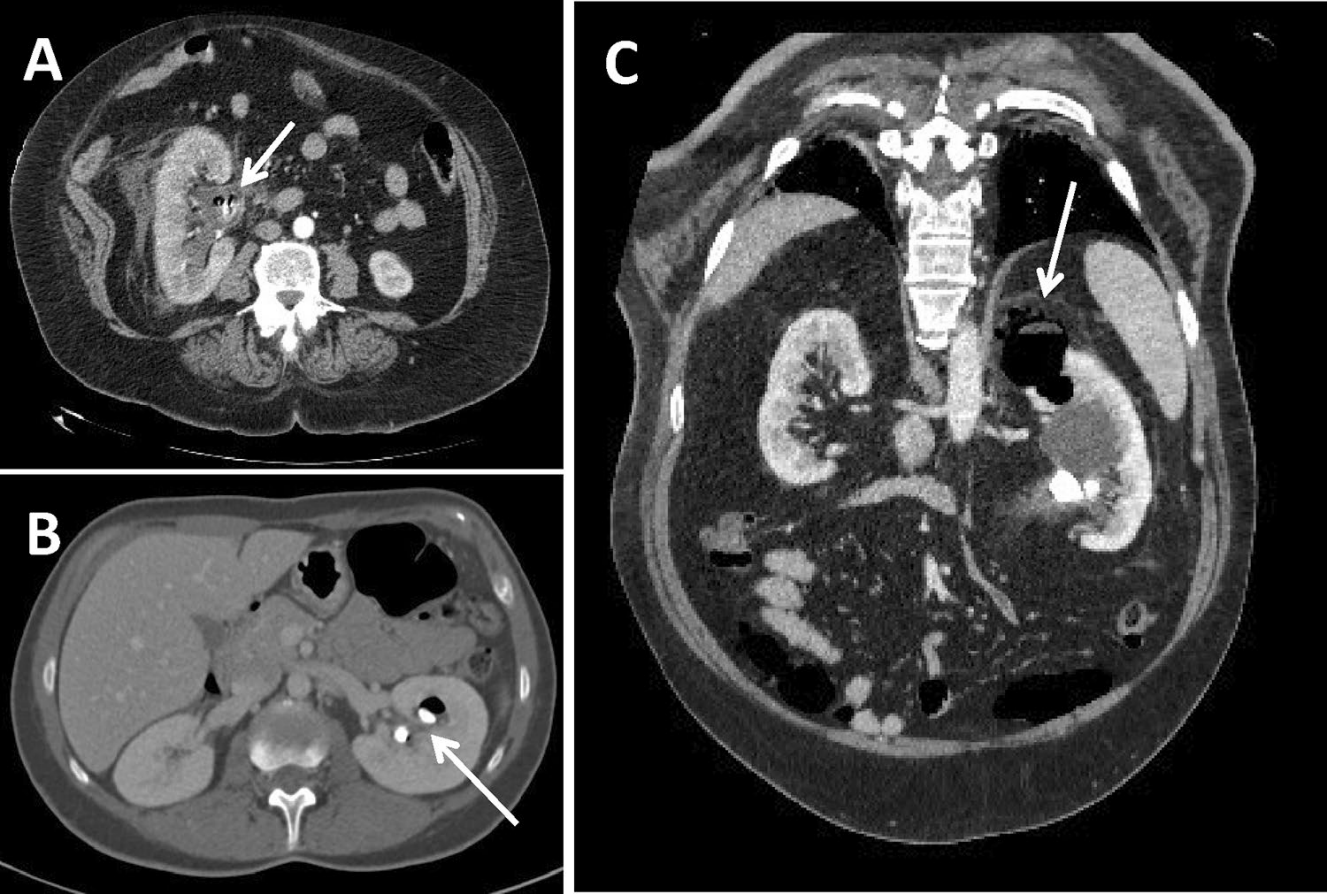
CT staging of emphysematous pyelonephritis. **a** Axial oblique MPR reconstruction of the parenchymal phase of a 53-year-old diabetic female patient presenting with mild fever shows a class I EPN, characterized by gas only inside collecting system, in association with the presence of a calculus in the renal pelvis (arrow). **b** Axial scan of the parenchymal phase of a 73-year-old diabetic female patient presenting with right flank discomfort shows two calyceal calculi with initial parenchyma spread of gas (arrow) in a class II EPN. **c** Coronal oblique MPR reconstruction of a 66-year-old diabetic female patient presenting with abdominal pain shows a class III EPN with a voluminous hypodense fluid-gaseous abscess filled with gas bubbles, dissecting the interstitial space and invading the perinephric space (arrow)

Subsequently, Huang et al. [49] proposed four classes of EPN based on CT scan, also providing a prognostic stratification, essential for patient’s management (Table 3). In particular, in class 1 gas is present only in the collecting system (Fig. 5a), in class 2 gas is present in the renal parenchyma without extension to the extrarenal area (Fig. 5b), in class 3A gas and or abscess are present in the perinephric area (Fig. 5c), in class 3B gas or abscess are present in the pararenal space, while in class 4 EPN is bilateral. Although no significant differences were noted in clinical features among the 4 classes, mortality progressively increases, with class 4 being the worst. In particular, class 1 and 2 patients can be treated with percutaneous drainage combined with antibiotics, as well as patients with more extensive EPN, such as class 3A and 3B, on condition there are < 2 risk factors (i.e., thrombocytopenia, acute renal function impairment, disturbance of consciousness, and shock). Nephrectomy should be promptly performed in class 3A and 3B with > 2 risk factors and in class 4 cases; however, given the instability of class 4 patients, bilateral percutaneous drainage should be tried first, in order to avoid an emergency nephrectomy [53].

**Table 3:** CT staging of emphysematous pyelonephritis

Classification	Class
Wan et al. (39)	I. Parenchymal destruction with either an absence of fluid collection or a presence of steaky or mottled gas
	II. Renal or perinephric fluid collection with bubbly or located gas or gas in the collecting system
Huang and Tseng (40)	1. Gas in collecting system only
	2. Parenchymal gas only
	3A. Extension of gas into perinephric space
	3B. Extension of gas into pararenal space
	4. EPN in solitary kidney or bilateral disease

### Differential diagnosis with other causes of intrarenal gas

**Fig. 6:**
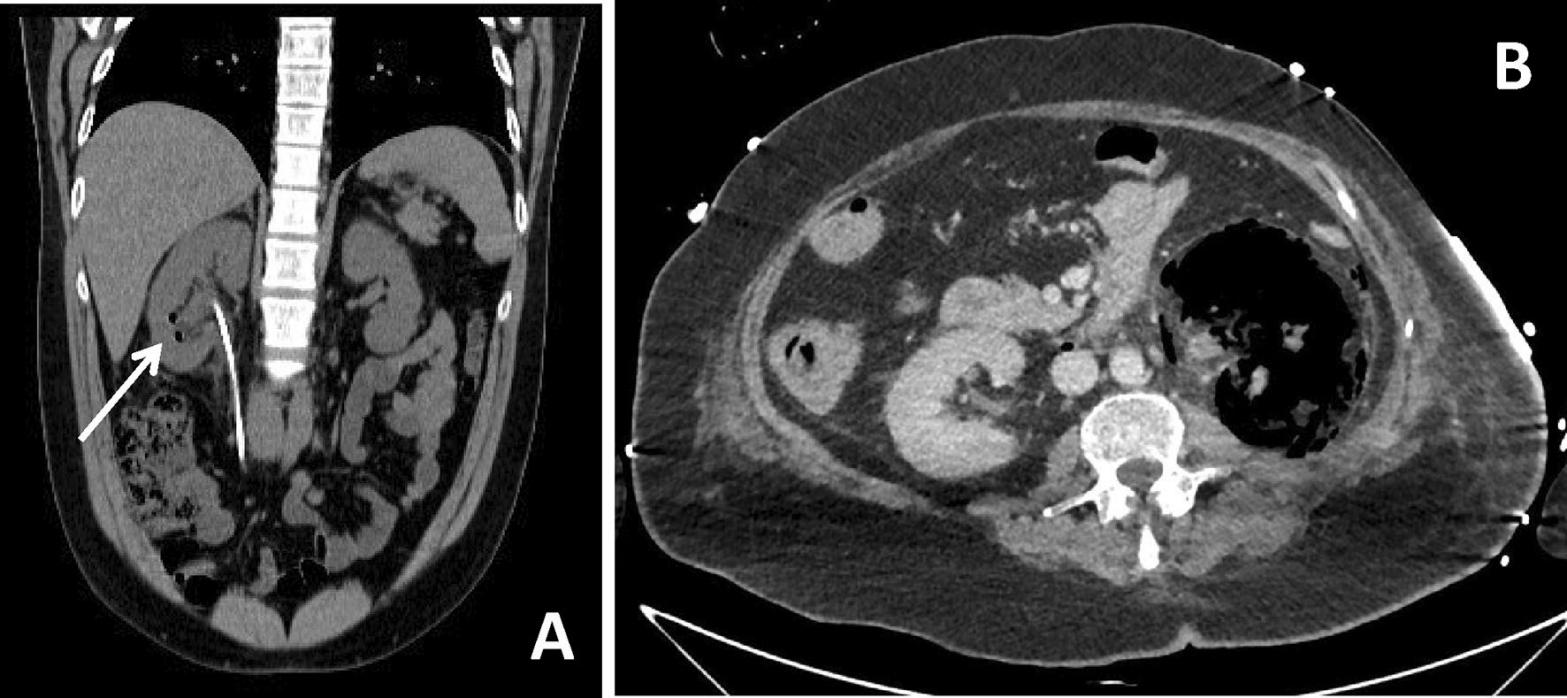
Pitfalls in renal gas collections diagnosis. **a** Coronal oblique MPR reconstruction of unenhanced phase of a 60-year-old male asymptomatic patient shows gas bubbles in the inferior calyces of right kidney (arrow) in patients with ureteral stent. **b** Axial scan of the parenchymal phase of a 56-year-old female patient presenting with abdominal pain, fever and hematuria shows unusual and severe case of an extensive renal infarction, where the left kidney is characterized by a widespread subversion of the parenchyma, which appears to have a mainly gaseous density, with concomitant signs of free air in the retroperitoneum

It is important to remember that a finding of gas in renal parenchyma can also be the result of an interventional procedure, such as biopsies, stent insertion (Fig. 6a), embolization or ablation, thus not always representing an infection [54, 55]. Failure to recognize the possibility of intrarenal gas after surgical procedures could lead to incorrect diagnoses and unrequited treatment.

Moreover, other than EPN, gas formation in the kidney is rare, but it has been described following spontaneous tumor cell necrosis in renal carcinomas [56] and after a massive hemorrhagic infarction due to the thrombosis of a renal artery [57]. In particular, similarly to the gas formation in intrauterine fetal demise, CO_2_ formation from anaerobe reticulocyte metabolism or O_2_ simple liberation from oxyhemoglobin is being considered as pathogenetic etiology for the intrarenal gas formation, with the latter especially enhanced in the case of hypervascularized malignant renal tissues [56]. For these reasons, the presence of gas in the kidney is not necessarily due to the formation of an abscess, but it can also be a feature of an extensive renal infarction (Fig. 6b), especially in those cases of hypervascularized tissues, such as renal tumors.

## Conclusion

Nephrolithiasis is a very common disease and, if the causal factors at its source are not resolved, can become chronic, predisposing and promoting the development of a pyelonephritis. In high-risk populations, like diabetic or immunosuppressed patients, infection can become complicated and lead to a XGP or an EPN, further reducing the prognosis of these already debilitated patients. Therefore, a possible diagnosis of XGP or EPN must always be taken into account when a pyelonephritis is associated with untreated kidney stones, especially whenever clinical presentation is atypical, current therapy is not effective and/or imaging shows features of dubious interpretation. Taking into account the data collected in our study, it is clear that these two entities still represent an important challenge for the radiologist. In fact, an unrecognized renal tumor can be hidden behind a suspected diagnosis of XGP or a suspected diagnosis of ENP can turn out to be a simple complication of an interventional procedure. Due to their rarity and atypical presentation, a multidisciplinary approach is required and an expert radiologist represents a key figure in the multidisciplinary team as he can help to differentiate between benign and malignant lesions and thus avoid unnecessary radical pre-diagnostic surgical procedures. In conclusion, this pictorial essay raises awareness on complicated pyelonephritis associated with chronic nephrolithiasis, in order to improve the diagnostic process and thus direct the affected patients toward correct management.

## Data Availability

Not applicable.
